# Prognostic Value of Pulmonary Vascular Resistance by Magnetic Resonance in Systolic Heart Failure

**DOI:** 10.5935/abc.20160020

**Published:** 2016-03

**Authors:** Óscar Fabregat-Andrés, Jordi Estornell-Erill, Francisco Ridocci-Soriano, José Leandro Pérez-Boscá, Pilar García-González, Rafael Payá-Serrano, Salvador Morell, Julio Cortijo

**Affiliations:** 1 Departamento de Cardiologia - Hospital General Universitario de Valencia, Valencia - Spain; 2 Fundación para la Investigación - Hospital General Universitario de Valencia, Valencia - Spain; 3 Unidad de Imagen Cardiaca - ERESA - Hospital General Universitario de Valencia, Valencia - Spain; 4 Departamento de Medicina. Universitat de Valencia, Valencia - Spain; 5 Departamento de Farmacologia. Universitat de Valencia, Valencia - Spain

**Keywords:** Vascular Resistance, Hypertension, Pulmonary, Heart Failure, Prognosis, Magnetic Resonance Spectroscopy

## Abstract

**Background:**

Pulmonary hypertension is associated with poor prognosis in heart failure. However, non-invasive diagnosis is still challenging in clinical practice.

**Objective:**

We sought to assess the prognostic utility of non-invasive estimation of pulmonary vascular resistances (PVR) by cardiovascular magnetic resonance to predict adverse cardiovascular outcomes in heart failure with reduced ejection fraction (HFrEF).

**Methods:**

Prospective registry of patients with left ventricular ejection fraction (LVEF) < 40% and recently admitted for decompensated heart failure during three years. PVRwere calculated based on right ventricular ejection fraction and average velocity of the pulmonary artery estimated during cardiac magnetic resonance. Readmission for heart failure and all-cause mortality were considered as adverse events at follow-up.

**Results:**

105 patients (average LVEF 26.0 ±7.7%, ischemic etiology 43%) were included. Patients with adverse events at long-term follow-up had higher values of PVR (6.93 ± 1.9 vs. 4.6 ± 1.7estimated Wood Units (eWu), p < 0.001). In multivariate Cox regression analysis, PVR ≥ 5 eWu(cutoff value according to ROC curve) was independently associated with increased risk of adverse events at 9 months follow-up (HR2.98; 95% CI 1.12-7.88; p < 0.03).

**Conclusions:**

In patients with HFrEF, the presence of PVR ≥ 5.0 Wu is associated with significantly worse clinical outcome at follow-up. Non-invasive estimation of PVR by cardiac magnetic resonance might be useful for risk stratification in HFrEF, irrespective of etiology, presence of late gadolinium enhancement or LVEF.

## Introduction

The occurrence of pulmonary hypertension (PH) is considered an indicator of poor prognosis in the progression of chronic heart failure (HF) with reduced ejection fraction (HFrEF).^1-3^ Some patients, along with increased pulmonary venous pressures secondary to persistently high left ventricular end-diastolic pressures, also develop abnormalities in pulmonary arterial (PA) structure which leads to an increase in pulmonary vascular resistance (PVR).^4^ The presence of this pre-capillary contribution to PH was recently associated with worse prognosis in advanced HF.^5^

In clinical practice, estimation of systolic pulmonary arterial pressure (sPAP) and other parameters by Doppler echocardiography is widely used to identify PH in patients with HFrEF.^6-9^ Nonetheless, the inconsistency of these methods is well recognized, and right heart catheterization still remains the gold standard for establishing a diagnosis of PH, despite of radiation exposure and risks associated with invasive procedures.

Cardiovascular magnetic resonance (CMR), however, allows comprehensive non-invasive evaluation of anatomy and function of right ventricle as well as pulmonary artery. Furthermore, late gadolinium enhancement (LGE) assessment has become essential in risk stratification of patients with chronic HF.^10,11^ Based on accurate non-invasive methods for measurement of PVR previously reported,^12,13^ we have recently described the prognostic value of PVR in patients with heart failure admitted for acute decompensation.^14^ In this analysis, we focused on the group of patients with systolic dysfunction in order to assess if it preserves its prognostic utility in this context.

## Methods

### Patient population

We prospectively enrolled 105 consecutive patients (average age 65.7 ± 11.7 years, 72% male) referred to our cardiac imaging unit between March 2011 and April 2014. Of these patients, 84 come from our previous analysis in HF patients with both reduced and preserved ejection fraction.^14^ All patients were recently admitted for acute decompensated HF in different hospitals of the reference area and underwent a CMR under clinician criterion for the evaluation of chronic HF when they were stabilized, either during admission or within the first two weeks after discharge. Only were included in this analysis those patients with a left ventricular ejection fraction (LVEF) ≤ 40% estimated by CMR. Diagnosis of HF was achieved as recommended by current guidelines.^3,15^ Written informed consent was obtained before CMR in each patient.

### Clinical variables

Medical history was examined in all patients, recording cardiovascular risk factors and medication. Relevant blood tests values (hemoglobin and creatinine at admission) were also recorded as well as significant electrocardiographic parameters (duration of QRS complex, and the presence of atrial fibrillation or left bundle branch block).

### Coronary angiography

All patients underwent coronary angiography at our institution as referral hospital during current admission or previously. Data from coronariography were recorded to define ischemic etiology of HF according Felker et al.^16^ criteria: history of myocardial infarction or revascularization, ≥ 75% stenosis of left main or proximal left anterior descending artery, or ≥ 75% stenosis of two or more epicardial vessels.

### Echocardiography

Echocardiographic data for analysis were recorded from studies during admission. LVEF, left ventricular end-diastolic and end-systolic diameters, tricuspid annular plane systolic excursion, E/e' ratio and sPAP were examined, although LVEF was the only parameter recorded in medical history in all patients. The other variables were considered when available.

### Cardiac magnetic resonance

CMR was performed with a 1.5 T unit (Magnetom Sonata, Siemens, Erlangen, Germany). For cine imaging, breath-holding ECG-gated steady-state free precession (SSFP) sequences were used as normally to acquire long and short axis slices, and hence evaluate ventricular volumes and function. A standard 17-segmented cardiac-model was used for segmentation and assessing areas of LGE images,^17^ acquired after intravenous injection (0.15 mL/kg) of dimeglubine gadobenate 0.5 M. The areas of necrosis or fibrosis were assessed using inversion recovery-SSFP sequences (repetition time 2.9-3.9 ms, echo time 1.5-2.0 ms, flip angle 45-90º, slice thickness 6 mm with inter-slice gap 4mm, in-plane spatial resolution 1.5-2 mm, temporal resolution 35-45 ms) ten minutes after contrast administration adjusting the inversion time (between 250 to 300 ms generally) to null normal myocardium. Flow imaging was performed perpendicular to the PA trunk with a velocity-encoded gradient echo sequence using an upper velocity limit of 150 cm/s (or the minimum velocity without signal aliasing). Two double-oblique orthogonal views oriented along the main PA were acquired with SSFP cine sequence and used as the reference to prescribe the plane perpendicular to the PA trunk for the acquisition of phase-contrast images. These parameters were applied as usually: repetition time/echo time 5.9-7.5/3.1-6.5 ms, slice thickness 6 mm, in-plane resolution 1.5-3 mm, 20 reconstructed cardiac phases, and temporal resolution 55-105 ms.

**Figure 1:**
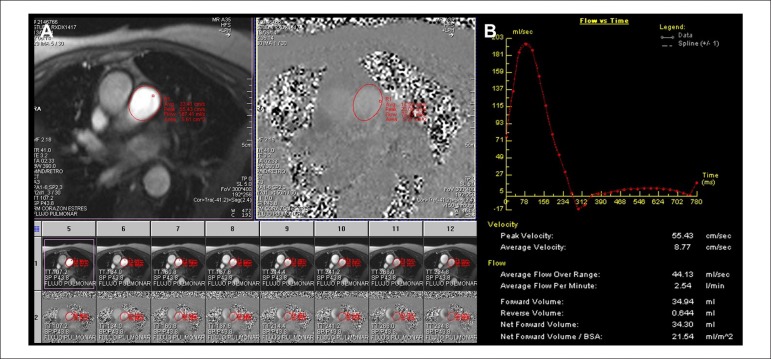
Cardiac magnetic resonance of a representative patient with high pulmonary vascular resistances. 72-year-old female patient with non-ischemic dilated cardiomyopathy and biventricular systolic dysfunction (left ventricular ejection fraction of 33% and right ventricular ejection fraction of 40%). (A) Phase-contrast images for quantification of pulmonary artery velocities. (B) Off-line analysis of pulmonary artery flow rate vs. time to calculate velocities and flows.

Images were analyzed by a single expert cardiologist in cardiac imaging using a specific software (Argus^®^, Siemens, Erlangen, Germany). Short axis slices were used to calculate ejection fractions and ventricular volumes using Simpson's method. LGE of the myocardium was visually identified by the CMR expert blinded to hemodynamic and echocardiographic data, considering both the presence (ischemic and non-ischemic patterns) as distribution of LGE (number of myocardial segments with LGE). PA cross-section were outlined in each cardiac phase to estimate PA area and flow, and calculate peak and average velocities during the complete cardiac cycle, minimum and maximum areas, and PA net forward volume (Figure 1). Ventricular volumes, ejection fractions and PA area were adjusted to body surface area.

PVR were calculated with this formula previously reported: PVR (in estimated Wood units [eWu]) = 19.38 - [4.62 x ln PA average velocity (in cm/s)] - [0.08 x right ventricular ejection fraction (RVEF) (in %)].^12^

### Clinical follow-up

Readmission for HF and all-cause mortality were considered as major adverse events at follow-up. Combination of both outcomes constitutes the primary endpoint. Data were collected from electronic centralized medical history, shared by all hospitals involved.

### Statistical analysis

Categorical values were expressed as absolute number and percentages, and continuous variables as mean ± standard deviation. Kolmogorov-Smirnov test was used for normality of the distribution. Patients were initially divided into tertiles according to the value of PVR on CMR. Comparisons between groups were made using analysis of variance (one-way ANOVA, with post-hoc multiple comparisons using Bonferroni test), and its prognostic role was assessed by construction of Kaplan-Meier survival curve. Subsequently, the sample was divided in two groups according to the optimal cut-off value of PVR calculated by receiver operating characteristic (ROC) curve to predict primary endpoint at follow up. Comparisons between both groups were made by Chi-Square test or unpaired t-Student test as appropriate.

A multivariate Cox regression model was performed with all variables with a p value < 0.10 in the univariate analysis to define the prognostic utility of PVR. Survival curves according PVR cut-off point were again constructed with the Kaplan-Meier method and compared by Log-rank test.

All tests were two-tailed and p value < 0.05 was considered statistically significant. Statistical analyses were performed using SPSS ^®^ software (version 17.0).

## Results

### Baseline patient characteristics according to PVR

Baseline characteristics of patients according to tertiles of PVR are presented in Table 1. We found a study population optimally medicated, with average LVEF by CMR 26.0 ± 7.7%, 43% with ischemic etiology of HF, and 29% of patients with atrial fibrillation. No differences were found in cardiovascular risk factors, medication, laboratory values or ECG parameters between different groups.

**Table 1:** General characteristics of patients according to tertiles of pulmonary vascular resistance by cardiac magnetic resonance

	**All (n = 105)**	**1^st^ tertile (PVR ≤ 4 Wu)**	**2^nd^ tertile (PVR > 4 ≤ 6 Wu)**	**3^rd^ tertile (PVR ≥ 6 Wu)**	**p value**
		**(n = 35)**	**(n = 35)**	**(n = 35)**	
Age	65.7 ± 11.7	67.2 ± 9.8	66.1 ± 11.8	63.8 ± 13.6	0.48
Male, n (%)	76 (73)	28 (80)	27 (77)	21 (60)	0.13
Hypertension, n (%)	72 (69)	22 (63)	25 (71)	25 (71)	0.79
Diabetes, n (%)	48 (46)	16 (46)	15 (43)	17 (48)	0.93
Dyslipidemia, n (%)	50 (48)	14 (40)	18 (52)	18 (52)	0.67
Smoking history, n (%)	76 (73)	28 (80)	26 (74)	22 (63)	0.42
Ischemic aethiology (%)	47 (43)	14 (40)	19 (56)	14 (40)	0.36
**Medication, n (%)**					
Betablockers	92 (88)	31 (88)	30 (85)	31 (88)	0.78
ACEI or ARBs	102 (97)	35 (100)	34 (97)	33 (94)	0.45
Diuretics	105 (100)	35 (100)	35 (100)	35 (100)	1.00
Aldosterone antagonists	55 (53)	16 (46)	16 (46)	23 (67)	0.18
Anticoagulants	24 (23)	7 (20)	7 (20)	10 (28)	0.45
**Blood values**					
Hemoglobin (g/dL)	12.8 ± 1.8	13.2 ± 1.6	12.5 ± 1.9	12.9 ± 1.8	0.44
Creatinine (g/dL)	1.09 ± 0.4	1.00 ± 0.2	1.15 ± 0.5	1.06 ± 0.3	0.17
**Electrocardiogram**					
Atrial fibrillation, n (%)	30 (29)	8 (23)	9 (25)	13 (37)	0.22
LBBB, n (%)	28 (26)	10 (40)	10 (28)	8 (23)	0.59
QRS complex (ms)	105.6 ± 25.5	105.3 ± 26.4	110.5 ± 25.1	101.5 ± 25.6	0.41

PVR: Pulmonary vascular resistance; ACEI: Angiotensin-converting-enzyme inhibitor; ARBs: Angiotensin II receptor blockers; LBBB: Left bundle branch block. Quantitative data expressed as mean ± standard deviation.

Worse ventricular function and higher ventricular diameters and volumes estimated by CMR were found in patients in upper tertiles (Table 2). A significant increase of sPAP by echocardiography was also observed in these patients (63.5 ± 14.6 mmHg in third tertile vs 38.6 ± 13.2 mmHg in first tertile, p = 0.03; based on available data).

**Table 2:** Echocardiography and cardiac magnetic resonance parameters according to tertiles of pulmonary vascular resistance

	**All (n = 105)**	**1^st^ tertile (PVR < 4 Wu)**	**2^nd^ tertile (PVR > 4 < 6 Wu)**	**3^rd^ tertile (PVR > 6 Wu)**	**p value**
		**(n = 35)**	**(n = 35)**	**(n = 35)**	
**Echocardiography**					
LVEF (%)	27.4 ± 10.9	27.7 ± 13.5	27.1 ± 10.5	26.8 ± 10.4	0.76
LVEDD (mm)^(a)^	60.3 ± 7.3	57.2 ± 5.0	59.1 ± 6.4	63.2 ± 8.2	0.10
LVESD (mm)^(b)^	48.3 ± 7.9	46.5 ± 5.9	47.8 ± 7.0	49.7 ± 9.8	0.52
TAPSE (mm)^(c)^	16.7 ± 5.1	19.0 ± 5.5	16.2 ± 5.3	16.6 ± 5.1	0.72
sPAP (mmHg)^(d)^	51.6 ± 13.7	38.6 ± 13.2	49.3± 12.2	63.5 ± 14.6	0.03
**Cardiac resonance**					
LVEF (%)	26.0 ± 7.7	30.0 ± 6.6	24.9 ± 8.1	23.1 ± 6.6	< 0.001
RVEF(%)	44.8 ± 17.2	55.6 ± 15.0	47.3 ± 12.1	31.5 ± 15.0	< 0.001
iLVEDV (%)	132.7 ± 39.5	122.0 ± 37.0	130.8 ± 38.8	145.3 ± 40.2	0.043
iLVESV (%)	98.2 ± 37.0	85.8 ± 33.4	99.0 ± 33.3	109.9 ± 40.8	0.023
iRVEDV (%)	71.7 ± 28.7	60.9 ± 26.1	71.8 ± 22.1	82.9 ± 33.2	0.007
iRVESV (%)	41.0 ± 23.7	28.1 ± 15.6	38.1 ± 15.5	56.6 ± 28.3	< 0.001
Presence of LGE, n (%)	67 (64)	18(51)	27 (77)	22 (63)	0.08
N° of segments with LGE	2.2 ± 2.3	2.2 ± 2.7	2.3 ± 1.9	2.1 ± 2.3	0.92
PVR (Wu)	5.42 ± 2.1	3.30 ± 0.9	5.19 ± 0.6	7.77 ± 1.4	< 0.001

LVEF and RVEF: Left and right ventricular ejection fraction; LVEDD and LVESD: Left ventricular end-diastolic and end-systolic diameters; TAPSE: Tricuspid annular plane systolic excursion; sPAP: Systolic pulmonary artery pressure; iLVEDV and iLVESV: Left ventricular end-diastolic and end-systolic volume indexed to body surface; iRVEDV and iRVESV: Right ventricular end-diastolic and end-systolic volume indexed to body surface; LGE: Late gadolinium enhancement; PVR: Pulmonary vascular resistance; Wu: Wood units; NS: No significant. Quantitative data expressed as mean ± standard deviation. *Available data from: (a) 71 patients (b) 65 patients (c) 31 patients (d) 48 patients.

### Prognostic impact of PVR estimated by CMR

**Figure 2:**
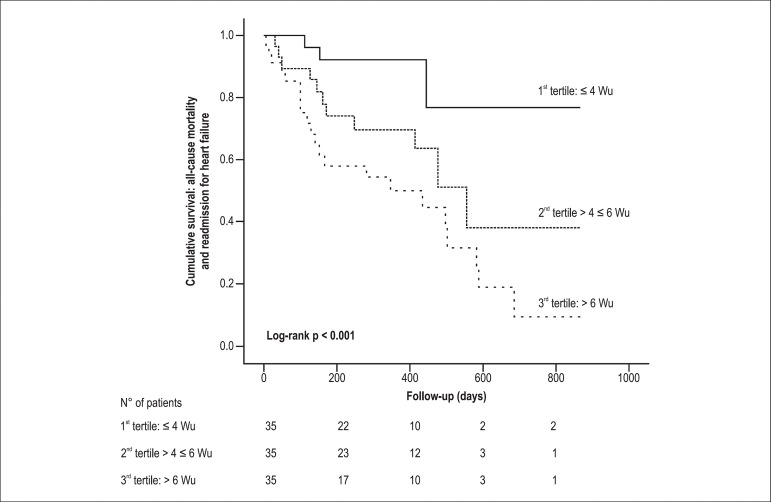
Pulmonary vascular resistance tertiles and clinical outcome. Kaplan-Meier survival curves according to tertiles of pulmonary vascular resistance estimated by cardiac magnetic resonance showing time to primary endpoint at follow up. Comparisons between groups were made using Log-rank test: p = 0.033 between first and second tertile, p < 0.001 between first and third tertile, and p = 0.106 between second and third tertile.

Patients with primary endpoint at long-term follow-up had higher values of PVR calculated by CMR (6.93 ± 1.9 vs. 4.6 ± 1.7 eWu, p < 0.001). When we analyze the probability of survival free of readmission for heart failure and all-cause mortality according to tertiles of PVR, patients in upper tertiles were significantly more likely to reach the composite adverse event (Figure 2).

### Univariate analysis

Univariate analysis of all cardiovascular risk factors and parameters of echocardiography and CMR are reported in Table 3. Thereby, univariate predictors (with p < 0.1) of primary endpoint included age, atrial fibrillation, left ventricular end-diastolic diameter by echocardiography, left and right ventricular end-diastolic volumes assessed by CMR, and PVR. Despite p value was above this limit, semiquantitative size of LGE (as measured by number of segments with LGE) was also included given its strong prognostic value in patients with HFrEF.

**Table 3:** Univariate analysis for total adverse events at follow-up

**Variable**	**HR (95% CI)**	**p value**
**Cardiovascular Risk Factors**		
Age	0.96 (0.91-1.01)	0.09
Male gender	1.75 (0.42-7.21)	0.43
Hypertension	1.37 (0.43-4.33)	0.59
Dyslipidemia	0.79 (0.26-2.39)	0.79
Diabetes	1.81 (0.61-5.35)	0.27
Ischemic etiology	2.32 (0.72-7.46)	0.15
Atrial fibrillation	6.58 (1.88-19.98)	0.003
**Echocardiography**		
LVEF (%)	0.81 (0.37-1.19)	0.12
LVEDD (mm)^(a)^	2.38 (1.03-5.46)	0.041
TAPSE (mm)^(b)^	0.99 (0.81-1.21)	0.94
PAPs (mmHg)^(c)^	1.03 (0.95-1.12)	0.43
**Cardiac Magnetic Resonance**		
LVEF (%)	1.01 (0.93-1.10)	0.74
iLVEDV (mL/m^2^)	1.02 (1.00-1.04)	0.020
RVEF (%)	1.00 (0.96-1.04)	0.78
iRVEDV (mL/m^2^)	0.97 (0.95-1.01)	0.08
PVR (uW)	2.31 (1.54-3.46)	< 0.001
Presence of LGE	1.31 (0.44-3.88)	0.61
Number of segments with LGE	1.11 (0.94-1.33)	0.21

LVEF and RVEF: Left and right ventricular ejection fraction; LVEDD: Left ventricular end-diastolic diameter; TAPSE: Tricuspid annular plane systolic excursion; sPAP: Systolic pulmonary artery pressure; iLVEDV and iRVEDV: Left and right ventricular end-diastolic volume indexed to body surface; PVR: Pulmonary vascular resistance; Wu: Wood units; LGE: Late gadolinium enhancement. *Available data from: (a) 71 patients (b) 31 patients (c) 48 patients.

### PVR ≥ 5 Wu as independent predictor of adverse outcome

In order to establish the optimal cutoff value of PVR to predict adverse events at follow-up, a ROC curve was carry out considering the primary endpoint as clinical outcome (PVR cut point: 5.0 eWu, area under the curve 0.81 [95% confidence interval 0.72-0.89], p < 0.001). General characteristics of both groups according to this cutoff value are summarized in Table 4. A higher prevalence of atrial fibrillation was observed when compared cardiovascular risk factors. As expected, patients with PVR ≥ 5.0 eWu had also worse biventricular systolic function and higher ventricular volumes, with a trend toward more frequent presence of LGE on CMR.

**Table 4:** General characteristics of patients according to optimal cutoff value of pulmonary vascular resistance to predict adverse events at follow-up

	**All (n=105)**	**PVR < 5.0 Wu (n=48)**	**PVR ≥ 5.0 Wu (n=57)**	**p value**
Age	65.7 ± 11.7	66.9 ± 9.9	64.6 ± 13.1	0.33
Male, n (%)	76 (72)	38 (79)	38 (67)	0.15
Hypertension, n (%)	71 (68)	32 (67)	39 (70)	0.58
Diabetes, n (%)	48 (46)	22 (46)	26 (46)	0.97
Dyslipidaemia, n (%)	51 (49)	23 (48)	28 (49)	0.90
Ischemic aethiology (%)	48(46)	21 (43)	27 (48)	0.64
Atrial fibrillation, n (%)	30 (29)	8 (16)	22 (38)	0.03
**Echocardiography**				
LVEF (%)	27.4 ± 10.9	30.7 ± 13.1	26.1 ± 9.7	0.10
LVEDD (mm)^(a)^	60.2 ± 7.2	56.8 ± 4.6	61.6 ± 7.6	0.01
LVESD (mm)^(b)^	48.3 ± 7.9	44.2 ± 6.3	49.8 ± 8.0	0.01
TAPSE (mm)^(c)^	16.9 ± 5.1	18.0 ± 4.9	16.5 ± 5.2	0.60
sPAP (mmHg)^(d)^	43.6 ± 13.7	39.3 ± 13.3	51.2 ± 14.3	0.13
**Cardiac magnetic resonance**				
LVEF (%)	26.0 ± 7.7	28.9 ± 5.4	22.8 ± 7.2	< 0.001
RVEF (%)	44.8 ± 17.2	54.5 ± 13.5	36.7 ± 15.7	< 0.001
iLVEDV (mL/m^2^)	132.7 ± 39.5	124.8 ± 35.3	139.4 ± 41.9	0.06
iLVESV (mL/m^2^)	98.3 ± 37.0	87.9 ± 31.4	107.0 ± 39.3	0.007
iRVEDV (mL/m^2^)	71.7 ± 28.7	62.3 ± 24.7	79.6 ± 29.5	0.001
iRVESV (mL/m^2^)	40.1 ± 23.7	29.4 ± 14.9	50.7 ± 25.4	< 0.001
Presence of LGE, n (%)	67 (64)	25 (52)	42 (73)	0.06
Number of segments with LGE	2.2 ± 2.3	2.1 ± 2.5	2.2 ± 2.1	0.78
PVR (Wu)	5.42 ± 2.1	3.64 ± 0.9	6.93 ± 1.5	< 0.001

LVEF and RVEF: Left and right ventricular ejection fraction; LVEDD and LVESD: Left ventricular end-diastolic and end-systolic diameters; TAPSE: Tricuspid annular plane systolic excursion; sPAP: Systolic pulmonary artery pressure; iLVEDV and iLVESV: Left ventricular end-diastolic and end-systolic volume indexed to body surface; iRVEDV and iRVESV: Right ventricular end-diastolic and end-systolic volume indexed to body surface; LGE: Late gadolinium enhancement; PVR: Pulmonary vascular resistance; Wu: Wood units; NS: No significant. Quantitative data expressed as mean ± standard deviation. *Available data from: (a) 71 patients (b) 65 patients (c) 31 patients (d) 48 patients.

To assess whether this cutoff value of PVR had an independent prognostic impact at follow-up, a Cox proportional hazard analysis was performed including all significant factors in univariate analysis (Table 5). After that, both PVR ≥ 5 eWu (HR 3.95; 95% CI 1.49-10.49; p = 0.006) and semiquantitative size of LGE (HR 1.18; 95% CI 1.01-1.37; p = 0.032) remained statistically significant.

**Table 5:** Multivariate Cox regression analysis

**Variable**	**HR (95% CI)**	**p value**
Age	1.00 (0.97-1.04)	0.87
Atrial fibrillation	1.51 (0.74-3.09)	0.25
iLVEDV (mL/m^2^)	1.01 (0.99-1.02)	0.43
iRVEDV (mL/m^2^)	1.01 (0.99-1.01)	0.40
Semiquantitative size LGE (nº segments)	1.18 (1.01-1.37)	0.032
PVR ≥ 5 Wu	3.95 (1.49-10.49)	0.006

iLVEDV and iRVEDV: Left and right ventricular end-diastolic volume indexed to body surface; LGE: Late gadolinium enhancement; PVR: Pulmonary vascular resistance; Wu: Wood units.

**Figure 3:**
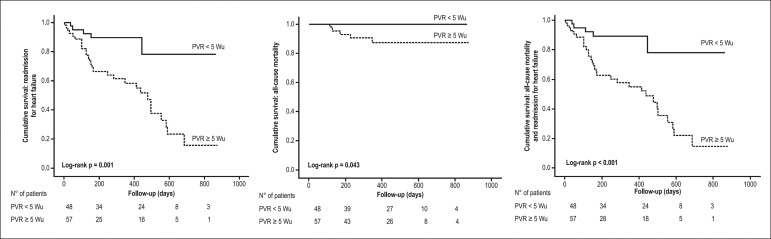
Pulmonary vascular resistance ≥ 5.0 eWu predicts worse prognosis. Kaplan-Meier curves showed time to adverse events (readmission for heart failure, all-cause mortality, and primary endpoint) according to optimal cutoff value of PVR.

At a mean follow-up of 9.1 (1-38) months, patients with PVR ≥ 5.0 eWu had a significantly worse prognosis, as indicated in Kaplan-Meier survival curves, both for readmission to HF (Log Rank test, p = 0.001) as for all-cause mortality (Log Rank test, p = 0.043) and risk to reach the primary endpoint (Log Rank test, p < 0.001) (Figure 3).

The main body of cardiac events during follow-up in these patients was represented by readmissions for HF, as follows: 5 readmissions for HF with no deaths in patients with lower PVR, and 28 readmissions for HF with 5 deaths in those with PVR ≥ 5.0 eWu. Of these total fatal events, 3 patients were previously admitted for acute decompensated HF.

## Discussion

Following the publication of the prognostic utility of PVR estimated by CMR to predict adverse events in chronic HF, the results of this study reinforce the prognostic value of this technique in the selected group of patients with systolic dysfunction. Thereby, we could observe that increased PVR by CMR remained as an independent predictor of worse prognosis at long-term follow up as well as semiquantitative size of LGE, and interestingly, irrespective of the presence of LGE. In clinical practice, routine use of this parameter could therefore provide additional valuable prognostic information for patients with HFrEF.

### Noninvasive diagnosis of PH

Diagnosis of PH in chronic HF remains challenging, because of inconsistency of echocardiography and risks derived from right heart catheterization, usually reserved for selected cases. Even so, in clinical practice, sPAP is often calculated by echocardiography from the velocity of the tricuspid regurgitant jet as an indirect estimation of the presence of PH, although as known remains a method with widely varying results and therefore unreliable in patients with suspected PH.

Other more accurate methods such as pulmonary artery acceleration time, right ventricular isovolumic relaxation time, or PVR itself, have been also described although systematically neglected in routine practice.^18-22^ Indeed, in our study with data recorded from real clinical practice, sPAP was calculated only in 48 of 105 patients, either because there was no significant tricuspid regurgitation or because inadequate visualization of right ventricle. This means, as shown, a major limitation of echocardiography.

A promising novel tool in this regard comes from CMR, which allows an accurate non-invasive estimation of PVR, as reported in previous studies. In our study, we employed the model proposed by Garcia-Alvarez et al.,^12^ using an equation with only two variables: RVEF and PA average velocity. This method showed a good limits of agreement with PVR quantified by right heart catheterization and allowed identify accurately those patients with increased PVR (considered as > 3 eWu). In addition, this model has also demonstrated its ability to monitor acute and chronic changes of PVR in a well-designed study that included: an experimental phase in pigs to evaluate acute changes after pulmonary embolization; serial changes in patients with chronic PH; and acute changes in PVR during vasodilator testing.^23^ This capability could therefore be valuable to noninvasive assessment and follow-up of patients with PH.

### Prognostic utility of incorporating PVR on CMR protocol

In patients with HFrEF, in which CMR is routinely used to define aetiology and clinical management, regular inclusion of PVR measurement could provide additional prognostic information in this respect, as previously described.^14^ In order to confirm the potential prognostic role of PVR in the group of patients with reduced LVEF, those referred to our cardiac imaging unit were long-term followed. We found that optimally medicated patients with increased PVR, according to optimal value calculated with ROC curve, had worse left and right ventricular systolic function and higher ventricular volumes, and showed an increased risk to achieve the primary endpoint at follow-up. This incremental risk was tested in univariate and multivariate analyses with other well-known prognostic factors such as LVEF, RVEF, presence and size of LGE or atrial fibrillation, and PVR remained as a solid predictor.

Although prognostic relevance of PH in chronic HF is well known,^24,25^ few studies have assessed the relationship between different PH subtypes and clinical outcomes. In this regard, the presence of an elevated transpulmonary gradient (> 12 mmHg) which reflects a significant contribution of pre-capillary component, appears to identify a subgroup of particular worse prognosis.^4,5^ This type of reactive PH is common among patients with acute decompensated HF, and therefore, taking into consideration the increased mortality rates observed in this subgroup of patients, it is essential to distinguish them at an early stage. Therefore, non-invasive estimation of PVR by CMR could emerge as a novel clinical tool in this context.

Since the majority of previous studies assessing the relationship between PH and adverse outcomes have normally used noninvasive parameters such as sPAP, the different contributions of pre- and post-capillary components could not be properly assessed so far.^26^ Both in our previous study^14^ as in this, we could indirectly evaluate the pre-capillary contribution to PH through the estimation of PVR which are closely related to increased pulmonary vascular tone. Thereby, we found that PVR by CMR were superior to predict adverse outcomes at long-term follow up when compared to sPAP by echocardiography and other consolidated risk factors such as LVEF, presence of LGE, or atrial fibrillation. As well as assessment of LGE on CMR has become an essential tool in the evaluation of HF patients in lasts years, among other variables with firmly established prognostic value such as LVEF, QRS duration or *New York Heart Association* functional class, our results suggest that inclusion of PVR measurement in standard CMR protocol could contribute to prognostic stratification of patients with HFrEF.

### Study limitations

The main limitation of the study is determined by the process of enrolling patients and subsequent analysis of clinical and echocardiographic data. Although inclusion was prospective, patients came referred from different centers to our cardiac imaging unit and therefore, there was no protocol on data record regarding blood tests, echocardiography or clinical management. This process caused data loss in some important echocardiographic parameters, and consequently were not included in univariate nor multivariate analysis, as indicated in methods.

In this regard, another important limitation comes from semiquantitative estimation of necrosis size by CMR. Thus, estimating the extent of LGE by number of segments and not by percentage with respect total myocardial mass probably conditioned the results of univariate analysis, knowing the solid prognostic value of LGE extent in this context. In order to minimize this issue, this variable was included in multivariate analysis despite being non-significant in the univariate.

Since right heart catheterization is still the reference test for diagnosis and follow-up of patients with PH, the absence of hemodynamic data could also be considered a limitation of the study.

Other important limitations are the reduced size of study population, the limited follow-up period and the fact of considering all-cause mortality, rather than cardiac mortality, as fatal event in the primary endpoint. Further studies will be therefore necessary to consolidate the prognostic value of PVR by CMR in patients with HFrEF.

## Conclusions

In patients with HFrEF, the presence of PVR ≥ 5.0 eWu on CMR is associated with significantly worse clinical outcome, considering both readmission for HF and all-cause mortality. Non-invasive estimation of PVR by CMR might be useful for risk stratification in HFrEF, irrespective of etiology, presence of LGE or LVEF.
